# Premature senescence of placental decidua cells as a possible cause of miscarriage produced by mycophenolic acid

**DOI:** 10.1186/s12929-020-00704-4

**Published:** 2021-01-04

**Authors:** Paz de la Torre, Miguel Fernández-de la Torre, Ana I. Flores

**Affiliations:** 1 Grupo de Medicina Regenerativa, Instituto de Investigación Sanitaria Hospital 12 de Octubre (imas12), Avda. Cordoba s/n 28041, Madrid, Spain; 2 Grupo de Enfermedades Raras, Mitocondriales y Neuromusculares, Instituto de Investigación Sanitaria Hospital 12 de Octubre (imas12), Avda. Cordoba s/n 28041, Madrid, Spain

**Keywords:** Placenta, Decidua, Miscarriage, Mycophenolic acid, Immunosuppression, Cell cycle, Nucleolar stress, Senescence, Autophagy

## Abstract

**Background:**

Successful pregnancy is supported by a healthy maternal–fetal interface (i.e., the decidual tissues) which holds the conceptus and safeguards it against stressors from the beginning of pregnancy. Any disturbance of this interface can presumably lead to the loss of pregnancy. The use of the immunosuppressive drug mycophenolic acid (MPA) should be discontinued in pregnancy given its abortive and embryotoxic effects. Direct teratogenic effects have been observed in mammalian embryos cultured in MPA, but the underlying mechanisms of abortion by MPA are less understood.

**Methods:**

Decidual stromal cells isolated from human placentas are cultured in the presence of clinically relevant doses of MPA. Data regarding the effects of MPA on the proliferation and viability of decidua cultures are first analysed and then, molecular pathways contributing to these effects are unravelled.

**Results:**

MPA treatment of decidual stromal cells results in loss of proliferation capacity and a decrease in the viability of decidua cultures. The molecular pathways involved in the effects of MPA on decidual stromal cells are a reduction in pre-rRNA synthesis and subsequent disruption of the nucleolus. The nucleolar stress stabilizes p53, which in turn, leads to a p21–mediated cell cycle arrest in late S and G2 phases, preventing the progression of the decidua cells into the mitosis. Furthermore, MPA does not induce apoptosis but activate mechanisms of autophagy and senescence in decidual stromal cells.

**Conclusion:**

The irreversible growth arrest of decidua cells, whose role in the maintenance of the pregnancy microenvironment is known, may be one cause of miscarriage in MPA treated pregnant women.

## Background

The placenta is an indispensable organ during pregnancy performing functions of metabolic exchange and endocrine regulation between the mother and fetus. The development of placenta begins at the implantation of the blastocyst, and is delivered at birth. The placenta plays a key role in the maintenance of pregnancy, and as the fetus depends on it for essential developmental functions, normal placental structure and function is vital for healthy growth of the fetus.

The placenta has both embryonic and maternal components, and the maternal portion, the decidua, develops from the endometrial layer of the uterus. Decidualization, or transformation of the endometrium into decidua, begins in the late secretory phase of the menstrual cycle, and is the first stage for the successful establishment of pregnancy [1]. The decidua is composed of glands, immune cells, blood and lymph vessels, and decidual stromal cells. Once conception occurs, decidual cells become a sensor responsive to embryo signals in such a way that can support its further development or on the contrary, promote its active rejection [2]. This quality control, that begins immediately post-conception, is probably continued throughout the first trimester of pregnancy.

Implantation involves the acceptance of the trophoblast by the decidua. Decidual cells encapsulate and safeguard the conceptus against diverse stressors. Furthermore, decidual cells have been highlighted as the main immune modulator of the placenta, and are vital for the immune acceptance of the allogeneic fetus [3]. Approximately, one-third of pregnancies end in loss, and eighty percent of miscarriages occur during the first trimester [4]. Although 50% of early pregnancy failures are caused by fetal malformations [5], there is a part of the remaining first-trimester miscarriages that could be attributed to a defective development of the decidua. Sonographic images have revealed some differences in first trimester decidua thickness in miscarried pregnancies compared to normal pregnancies [6].

Mycophenolic acid (MPA) is a powerful immunosuppressive drug currently used to prevent graft rejection after transplantation of organs such as kidney, liver and heart [7–9]. MPA is also used to control inflammation in autoimmune conditions including lupus, vasculitis, rheumatoid arthritis, and psoriasis [10], as well as in neurological diseases such as myasthenia gravis, multiple sclerosis, dysimmune neuropathies, and inflammatory myopathies [11]. The antitumoral effect of MPA has also been described [12]. MPA blocks the de novo purine biosynthesis by a reversible, noncompetitive inhibition of the enzyme inosine-5′-monophosphate dehydrogenase (IMPDH). The inhibition of IMPDH in lymphocytes causes a reduction in the guanine nucleotide pool, and as a result prevents lymphocyte proliferation. IMPDH is ubiquitously expressed and a similar cytostatic effect has been reported on other cell types such as fibroblasts [13] endothelial cells [14], smooth muscle cells [15], or cardiac stem cells [16].

The use of MPA during pregnancy has been associated with an increased risk of miscarriage in the first trimester [17], with up to 45–49% of spontaneous abortions in pregnant women exposed to mycophenolate mofetil, a prodrug of MPA, having been reported as compared to 12–33% under other immunosuppressive treatments. Furthermore, an estimated 22–26% rate of congenital defects has been described in live births with mycophenolate mofetil exposures during pregnancy [18], mainly responding to a pattern known as EMFO tetrad [19], i.e. “Ear (microtia and auditory canal atresia), Mouth (cleft palate and lip), Fingers (brachydactyly of the 5th fingers and hypoplastic toenails), and Organs (heart, kidney, central nervous system, diaphragm and eye)”.

In the present study, we have raised the question of how MPA may cause spontaneous abortion. Although it is known that a very considerable amount of mycophenolate crosses the placental barrier [20], which has a direct effect on the fetus, to the best of our knowledge there is no study evaluating whether placental cells are susceptible to the stressful effects of MPA. This work presents the in vitro effects of MPA treatment on the biology of stromal cells of the decidua and discusses how this could alter the placental function in the early stages of pregnancy.

## Material and methods

### Decidua cells isolation

Human placentas were obtained during natural or cesarean births at the Department of Obstetrics and Gynecology under written informed consent approved by the Ethics Committee from Hospital Universitario 12 de Octubre. Cells were isolated and cultured from placental membranes as described previously [21]. Briefly, placental tissue was digested with trypsin (Gibco, Thermo Fisher Scientific, Madrid, Spain), and isolated cells were seeded at 1.16 × 10^5^ cells/cm^2^ and cultured in Dulbecco Modified Eagle Medium (DMEM) supplemented with 2 mM glutamine, 0.1 mM sodium pyruvate, 55 μM β-mercaptoethanol, 1% non-essential amino acids, 1% penicillin/streptomycin, 10% fetal bovine serum and 10 ng/ml epidermal growth factor (EGF) (Sigma-Aldrich Química SA, Madrid, Spain). Non-adherent cells were removed and the karyotype analysis, fluorescence in situ hybridization (FISH), and short tandem repeats (STR) analysis showed that the cell population attached to the plastic was of maternal origin, and therefore, from the decidua [21]. Our group characterized these decidual cells as mesenchymal-like cells [21] and named them as decidua mesenchymal stromal cells (DMSC).

### Mycophenolic acid (MPA) treatment

Mycophenolic acid (Sigma-Aldrich Quimica SA, Madrid, Spain) was reconstituted in methanol at 50 mg/ml and diluted in phosphate-buffered saline (PBS) before use. Cells were treated with either methanol used as vehicle or MPA, as indicated. The dose used was 10 μg/ml, which is in the range of those clinically achieved in the plasma of MPA-treated transplanted patients [22, 23].

### Cell viability assays

Cell death was evaluated by the FITC Annexin V Apoptosis Detection Kit (BD Biosciences, CA, USA) following the manufacturer’s protocol and analyzed by a FACScalibur flow cytometer (Becton–Dickinson Immunocytometry, Mountain View, CA, USA). Cell survival was determined by Alamar blue assay according to the manufacturer’s instructions (Invitrogen, Thermo Fisher Scientific, Madrid, Spain). After MPA treatment, the culture medium was replaced by complete DMEM containing 10% of Alamar blue reagent, cells were incubated for 90 min at 37 °C to allow viable cells to convert resazurin (blue) into resorufin (purple) and the fluorescence signal was measured at excitation/emission wavelengths of 530/590 nm on a multi-modal plate reader (2300 Enspire, PerkinElmer España S.L., Madrid, Spain).

### Proliferation assay

Cell proliferation was quantified by a colorimetric immunoassay based on the incorporation of a thymidine analogue, 5-bromo-2-deoxyuridine (BrdU) to DNA, and according to the manufacturer’s instructions (Roche Diagnostics, Barcelona, Spain). BrdU was added to the cell culture and cells were incubated for 24 h. Cells were fixed and DNA was denatured so BrdU was accessible to a specific antibody conjugated with peroxidase. Subsequently, a peroxidase substrate solution was added, and the reaction product was detected at 370 nm by a multi-modal plate reader (2300 Enspire, PerkinElmer España S.L., Madrid, Spain).

### Cell cycle analysis by propidium iodide (PI) staining

DMSC were seeded in a 6-well plate and the next day, MPA was added. Following 48 h, cells were harvested, fixed in cold 70% ethanol, spun down, rinsed once with PBS, and suspended in 0.5 ml of FxCycle PI/RNase Staining Solution (Molecular Probes, Thermo Fisher Scientific, Madrid, Spain). DNA fluorescence of 2 × 10^5^ cells was measured by flow cytometry analysis (FACS) using a FACScalibur flow cytometer (Becton–Dickinson Immunocytometry, Mountain View, CA, USA). Cells in G0/G1 and G2/M phases of the cell cycle were determined using the BD CellQuest Pro software.

### Immunofluorescence assay

Cells were plated on Lab-Tek chamber slides (Nunc, Thermo Fisher Scientific, Madrid, Spain) at a density of 1 × 10^5^ cells/cm^2^. After MPA treatment, cells were fixed with 10% formalin for 10 min at room temperature, and permeabilized with 0.3% Triton-X 100 for 10 min. Non-specific binding was blocked with 5% horse serum. Mouse monoclonal antibody for human nucleophosmin or B23 (Santa Cruz Biotechnology Inc, TX, USA) was incubated overnight at 4 °C, rinsed with PBS twice and incubated with fluorescein isothiocyanate (FITC)-conjugated secondary antibody (Jackson Immunoresearch Laboratories, Vitro SA, Madrid, Spain). Nuclei were counterstained with 0.2 mg/ml 4′,6′diamidino-2-phenylindole (DAPI) (Sigma-Aldrich Quimica SA, Madrid, Spain) for 1 min and visualized by a Zeiss LSM 510 Meta Inverted Confocal Microscope (Carl Zeiss Meditec Iberia SAU, Madrid, Spain). B23 signal localized in nucleolar bodies was analyzed using the ImageJ software and expressed as Corrected Total Cell Fluorescence (CTCF).

### Real-time quantitative PCR (RT-qPCR)

Relative expression of the ribosomal precursor 45S with respect to the expression of the housekeeping gene β-actin was assessed. Total RNA was recovered from silica membrane columns of NZY Total RNA Isolation kit (NZYTech, Lda., Lisboa, Portugal) after DNAse I digestion. High Capacity cDNA Reverse Transcription kit was used to reverse transcribe the RNA (Applied Biosystem, Thermo Fisher Scientific, Madrid, Spain). Specific primers and length of products to human 45S and β-actin are shown in Table 1. Real-time PCR was performed using an ABI 7500 fast sequence detection system (AppliedBiosystems, Thermo Fisher Scientific, Madrid, Spain) and SYBR green qPCR Master mix (Promega Biotech Ibérica SL). Data were analyzed using the 2^−ddCt^ method of relative gene expression, with the target gene values normalized to the housekeeping gene β-actin.

**Table 1:** Real-time quantitative PCR primers and length of products

β-actina	Forward sequence (5′ to 3′): AGAGCTACGAGCTGCCTGAC Reverse sequence (5′ to 3′): AGCACTGTGTTGGCGTACAG	84 bp
45 S	Forward sequence (5′ to 3′): CGTGGTGTGAAACCTTCCGA Reverse sequence (5′ to 3′): CCCAAGAGGAGAGGGGGTT	91 bp

### Western blot assay

Lysis buffer containing 1% NP-40 was used to extract cellular proteins. Fifteen micrograms of protein were separated into 10% or 4–20% SDS-PAGE polyacrylamide gels (Bio—Rad Laboratories SA, Madrid, Spain). PVDF membranes (Bio Rad Laboratories SA, Madrid, Spain) were blocked using 10% BSA in Tris-buffered saline with 0.1% Tween 20 (TBST) for 1 h, and hybridized with primary antibodies for: p53 (rabbit anti-human p53 monoclonal antibody; 1:1000) (Cell Signaling Technology Inc, MA, USA), LC3II (rabbit anti-human; 1:1000) (Sigma-Aldrich Quimica SA, Madrid, Spain), p21 (rabbit polyclonal antibody; 1:500) (Proteintech, Manchester, UK), p62 (rabbit anti-human; 1:10,000) (Cell Signaling Technology Inc, MA, USA), Phospho-mTOR (Ser2448) (rabbit anti-human; 1:1000))(Cell Signaling Technology Inc, MA, USA), Phospho-p70 S6 Kinase (Thr389) (rabbit anti-human; 1:1000) (Cell Signaling Technology Inc, MA, USA), or α-tubulin (goat anti-mouse; 1:1000) (Abcam, Cambridge, UK) overnight at 4 °C. The membranes were incubated with secondary antibodies (Santa Cruz Biotechnology Inc, TX, USA) for 1 h at room temperature. Protein bands were visualized by enhanced chemiluminescence detection reagents (Bio-Rad Laboratories SA, Madrid, Spain). Tubulin was used as the loading control. All experiments were performed in triplicate.

### Senescence-associated beta galactosidase assay (SA-β-gal)

SA-β-gal assay was performed as described previously [24]. Briefly, cells were fixed for 15 min, washed with PBS, stained with SA-β-gal solution overnight at 37 °C, and visualized and imaged under a Leica DMIL microscope (Leica Microsistemas S.L.U., L’Hospitalet de Llobregat, Spain).

### Statistical analysis

Student t-tests were done using GraphPad software. The results were considered statistically significant when P values were less than 0.05.

## Results

### Antiproliferative effect of MPA on DMSC

**Fig. 1:**
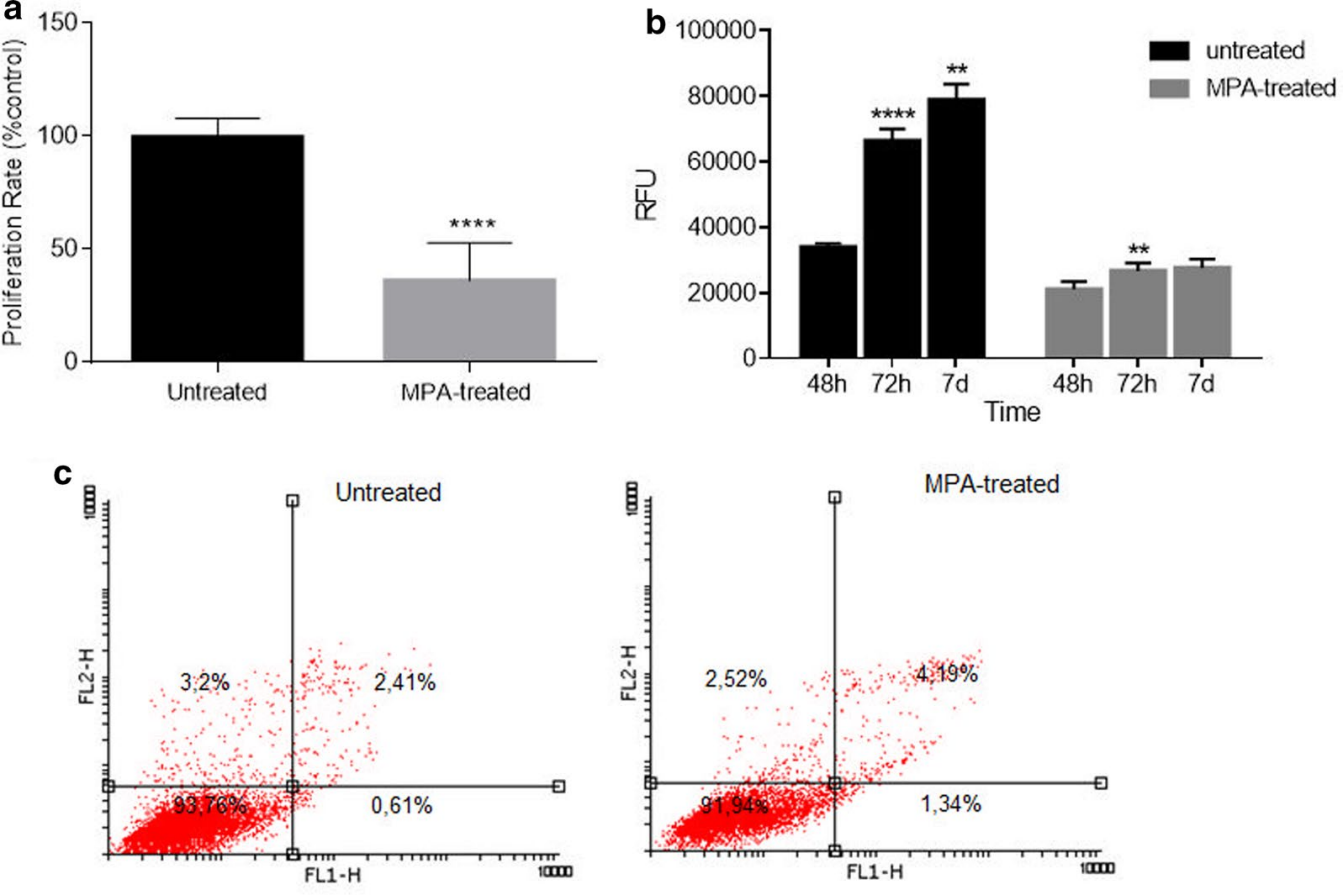
**a** Anti-proliferative effect of MPA on DMSC. DMSC were exposed for 48 h to MPA and proliferation measured by 5-Br-deoxyuridine incorporation. Mean values and SD were calculated from three independent experiments measured in sixfold each and expressed as a percentage with respect to untreated controls. (****P ≥ 0.0001). **b** Viability of untreated or MPA-treated DMSC from 48 h, 72 h and 7 days measured by alamar blue assay. Mean values with SD shown of DMSC cultures measured fivefold. Data in each condition, untreated or treated, are compared with respect to the immediately previous time (**P ≥ 0.01****P ≥ 0.0001). **c** Apoptosis/necrosis flow cytometry analysis of untreated DMSC (left) or treated for 48 h with MPA (right) showing viable cells (lower left quadrant), early apoptotic cells (lower right quadrant) and late apoptotic/necrotic cells (upper right quadrant). No apoptotic effect of MPA was found

The immunosuppressive properties of MPA rely on its antiproliferative effects on the T and B lymphocyte populations. To assess the potential antiproliferative effect of MPA on DMSC, cultured cells were treated with MPA for 48 h As Fig. 1a shows, proliferation rate of treated cells, measured by the incorporation of the thymidine analogue BrdU to newly synthetized DNA, was significantly reduced when compared to untreated cells. This antiproliferative effect obviously led to a reduction in the final number of cells in the culture after 48 h of MPA treatment with respect to untreated, as observed under the microscope (data not shown). To quantify the effects of MPA on DMSC viability over time, Alamar blue assay was used based on the reducing power of living cells (Fig. 1b). As expected, untreated cultures grew over time and the number of viable cells continued to rise significantly from 48 h until 7 days, although the growth rate between 72 h and 7 days was slower, probably because the culture was near to confluency. On the other hand, MPA treated cultures presented a significantly lower growth with respect to untreated, and with near absent growth from 48 h to 7 days. To exclude cytotoxic effects of MPA on DMSC, flow cytometer analysis of annexin V/propidium iodide stained cells was realized. As Fig. 1c shows, DMSC treated with MPA for 48 h were mainly live cells, and there was no relevant increase in the number of apoptotic or necrotic cells with respect to untreated cells.

### Cell cycle arrest in late-S and G2/M

**Fig. 2:**
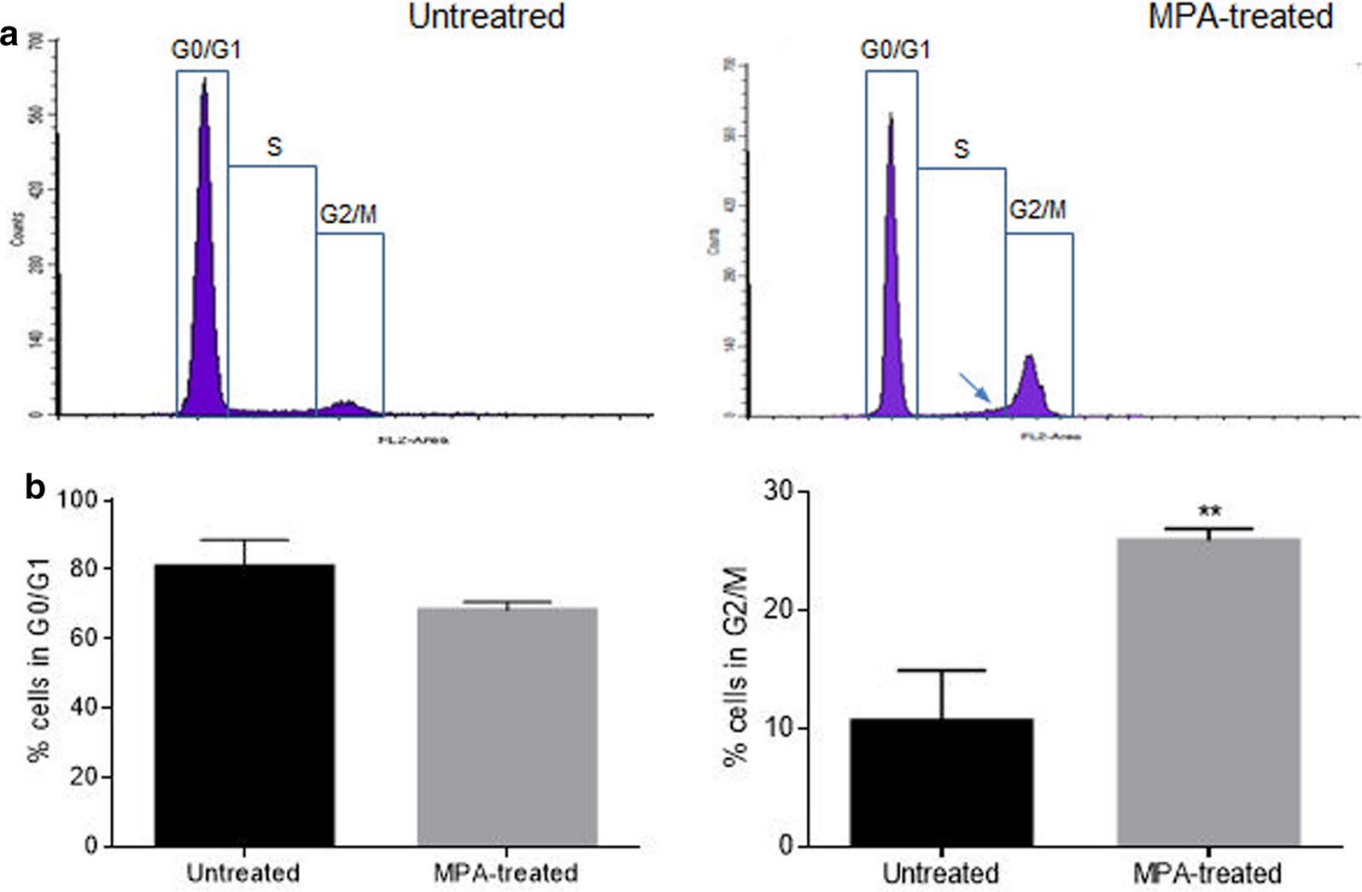
Cell cycle arrest in late-S and G2/M in MPA-treated cells. DMSC were treated with MPA for 48 h and then incubated with propidium iodide (PI) and RNase for 15 min. **a** Fluorescence histograms obtained by flow cytometry analysis of stained cells: Y-axis gives the number of cells, and the X-axis gives PI fluorescence intensity, which is proportional to DNA content. Cells treated with MPA tended to be retained in the late S phase (arrow) as well as being arrested in G2/M (representative image of three experiments). **b** Comparison of the percentages of cells in gated areas corresponding to G0/G1 and G2/M in untreated and MPA-treated cells (n = 3) (**P ≥ 0.01)

To elucidate the phase of cell division which is arrested by MPA, cell cycle progression was analysed using propidium iodide staining and flow cytometry analysis. Fluorescence intensity of propidium iodide is proportional to the amount of DNA in the cell, and results in two histogram peaks corresponding to G0/G1 and G2/M phases separated by the S phase plateau. Histograms from untreated cells and cells exposed to MPA for 48 h were compared and several differences were evident. In MPA-treated cells, certain G0/G1 cell population shifted to G2/M and there was a small retention of cells in late-S (Fig. 2a)*.* Therefore, the percentage of cells in the G2/M phase in treated cells was 25.90 ± 0.99%, significantly higher than the 10.63 ± 4.3% found in untreated samples (Fig. 2b). The increase in the MPA treated cells of the cell population at the G2/M phase was, in part, at the expense of a decrease in the percentage of total cell population in the G0/G1, 68.18 ± 2.44% in treated cells, and 80.85 ± 7.6% in untreated samples (Fig. 2b). Furthermore, the change in the distribution of cells in S phase was noticeable with most cells accumulated in late-S which means a blockade for cell to entry into mitosis (Fig. 2a).

### MPA strongly stabilizes p53 protein and the downstream effector p21

**Fig. 3:**
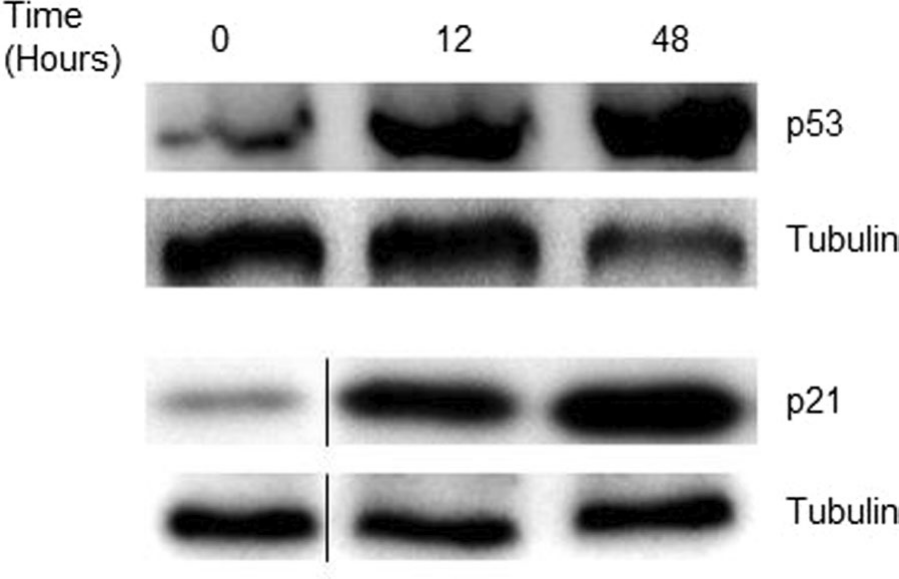
Induction of p53 and p21 proteins in DMSC exposed to MPA. Protein homogenates were subjected to western blot analysis for p53 and p21 analysis. The thin black line in p21 blot indicates that the lanes were run on the same gel but were noncontiguous. Tubulin protein was used as loading control

The arrest of the cell cycle is a common cellular response to diverse stressful conditions, DNA damage, or failures during replication. Stopping the cell cycle, cells could activate mechanisms of recovery from damage before resuming normal proliferation, and the tumor suppressor p53 is often a key factor in this cell cycle control. Total lysates from untreated and MPA-treated DMSC were obtained and analyzed for the total amount of p53 protein. Western blot analysis showed that MPA treatment of DMSC for 12 and 48 h resulted in higher p53 levels than those that appear in untreated cells (Fig. 3).

The cyclin-dependent kinase inhibitor p21 is commonly implicated in p53-mediated cell cycle arrest [25, 26], therefore we assessed whether MPA-treated cells displayed increased p21 levels. Western blot analysis of the DMSC total lysates showed that p21 expression was strongly induced after 12 h and 48 h of MPA treatment (Fig. 3).

### MPA promotes nucleolar disintegration

The nucleolus is the subnuclear structure where the synthesis of ribosomal RNA and the assembly of ribosomes occur. Since most cellular stresses are associated with the disruption of nucleolar integrity, the nucleolus has gained attention as a cellular stress regulator and the concept of ‘nucleolar stress’ has arisen.

**Fig. 4:**
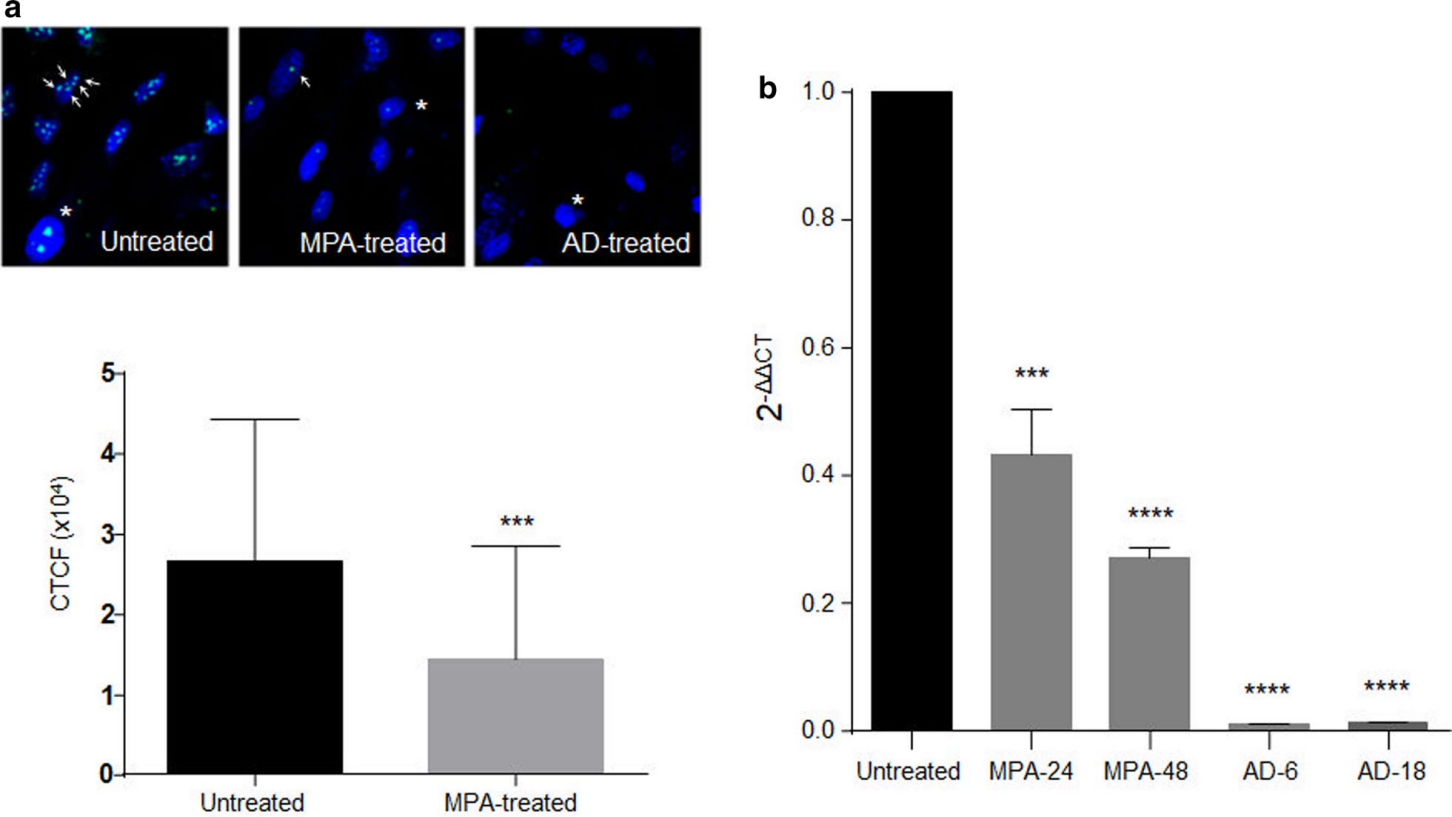
MPA treatment induces nucleolar stress in DMSC. **a** Confocal microscopy of nucleophosmin/B23 staining (arrows) in DMSC treated for 48 h with MPA showed reduced numbers and sizes of nucleoli (asterisks) when compared to untreated cells. Actinomycin D (AD) treatment for 18 h served as a positive control of disorganization of nucleoli. Nuclei were counterstained with DAPI. The quantification of total fluorescence by ImageJ was expressed as the corrected total cell fluorescence (CTCF) (***P ≥ 0.001). **b** Expression level of the ribosomal precursor 45S measured by quantitative PCR. MPA treatment downregulated transcription of the gene for 45S ribosomal precursor. Data were normalized with respect to β-actin gene expression and results are shown as relative expression to untreated cells (control) calculated by the 2^_ddCt_ method. As a positive control of nucleolar stress, actinomycin D (AD) was used (**P ≥ 0.01****P ≥ 0.0001)

We wanted to assess to what extent the treatment with MPA induces cellular stress in DMSC and thus, we searched for the presence of nucleolar stress indicators in MPA treated cells. Some described hallmarks of nucleolar stress are 1) reduction in nucleoli size and volume and 2) inhibition of rRNA transcription [27]. To have positive control of nucleolar disorganization we used 8 nM actinomycin D (AD), which at a low nanomolar dose acts selectively inhibiting Pol I and blocking ribosome biogenesis [28]. Accordingly, we treated DMSC with MPA or AD at different time points and analyzed the effects of both treatments. Protein B23 (also known as NPM1 and nucleophosmin) is the most abundant protein in the nucleolus and was used to detect the nucleoli in the cells. B23 immunofluorescence counterstained with DAPI revealed that the number and size of the nucleoli in MPA-treated cells were significantly smaller than in untreated cells (Fig. 4a). AD treatment of the cells resulted in almost complete disintegration of nucleoli. B23 immunostaining was quantified and the total B23 fluorescence was significantly lower in MPA-treated cells with respect to untreated cells (Fig. 4a). On the other hand, we used quantitative PCR to measure the transcriptional level for the rRNA 45S which is a precursor to rRNAs. Relative expression of 45S gene of treated cells with respect to untreated ones showed a significant decrease in the transcriptional level of the 45S gene as a consequence of MPA treatment for 24 h or 48 h (Fig. 4b). As expected, treatment with AD caused a strong reduction in the expression of the ribosomal precursor gene in DMSC.

### MPA induces autophagy in DMSC

Autophagy is a highly conserved catabolic pathway whereby cytoplasmic constituents, including organelles and long-lived proteins, are sequestered in a double membrane autophagic vacuole, (the autophagosome) and delivered to lysosomes for degradation. Autophagy is always present at a baseline level to ensure the turnover of cellular components and the maintenance of cellular homeostasis. Autophagic flux can be upregulated in response to various stressful conditions such as nutrient deprivation, reactive oxygen species, DNA damage, protein aggregates, damaged organelles, or intracellular pathogens [29]. In cells, LC3-II protein level is considered a good indicator of autophagy. During autophagy, the cytoplasmic soluble form of LC3 (or microtubule-associated protein 1A/1B-light chain 3)—LC3-I—is lipidated forming LC3-II that is recruited to the autophagosomal membranes. Following fusion of autophagosomes and lysosomes, autophagosomal components, including LC3-II, are degraded by lysosome hydroxylases [30].

**Fig. 5:**
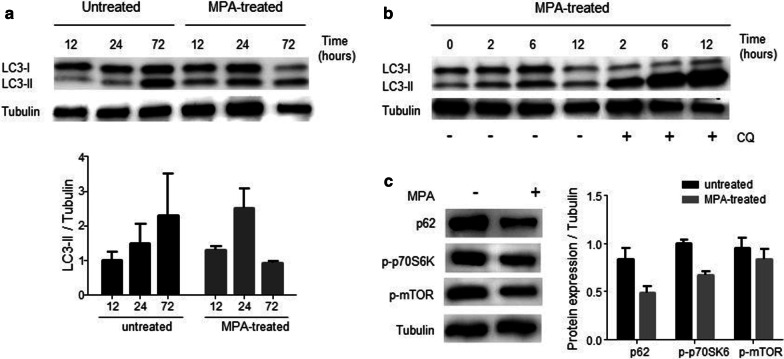
Autophagy markers induced in DMSC by MPA treatment. **a** Endogenous LC3-I and LC3-II levels in DMSC in untreated and after MPA treatment. DMSC were cultured for the indicated times, and total lysates subjected to immunoblot analysis using anti-LC3 antibody and anti-tubulin antibody. LC3-I and LC3-II positions are indicated. **b** To prevent lysosomal degradation of LC3-II, 30 µg/ml of chloroquine (CQ) was added to the medium where indicated. The increase in LC3-II as a consequence of the addition of the lysosomal inhibitor would indicate that the observed decrease in LC3-II in MPA treated DMSC would be due to an increased autophagic flux. **c** Lysates from MPA-treated cells for 72 h were subjected to immunoblot analysis for three indicators of autophagy: p62, whose accumulation is among the best characteristic of autophagy-deficient tissues, and the phosphorylated forms of mTOR and p70-S6K, which negatively regulate autophagy

To find out if MPA has any effect on autophagy mechanisms in DMSC, total protein extracts from MPA-treated or untreated cells were submitted to immunoblot to detect LC3-II protein. LC3 antibodies recognize both forms, the soluble LC3-I protein and the lipidated form LC3-II protein. The results showed that LC3-II levels were higher in MPA-treated samples than those in untreated samples after 12 or 24 h of treatment (Fig. 5a). In cells subjected to longer treatment (72 h), LC3-II levels notably increased with respect to the previous times in untreated cells, while in the MPA sample both LC3-I and LC3-II diminished. The decrease in LC3-I levels could suggest a high conversion into LC-3-II. With regard to LC3-II, this form is consumed in the autophagic process, and a very high autophagy activity could lead to rapid disappearance of LC3-II protein. To assess if MPA treatment is inducing a faster autophagic flux in DMSC, the lysosomal inhibitor chloroquine was added during the treatment and changes in LC3-II were evaluated. Chloroquine prevents the degradation of autophagosomes by neutralizing the lysosomal pH and in doing so, reveals the real quantity of autophagosomes in the cell at a fixed time. The difference in the amount of LC3-II between samples in the presence and absence of chloroquine represents the amount of LC3 that is delivered to lysosomes for degradation (i.e., autophagic flux). In this assay, MPA or MPA plus chloroquine were added to DMSC and culture for 2, 6, and 12 h (Fig. 5b). A longer use of chloroquine has been associated with loss of cell viability. Figure 5b shows that an increase in LC3-II levels is produced in the presence of chloroquine regarding MPA only, and this effect increases over time, which suggests that MPA treatment produces a progressive increase in the number of autophagosomes, i.e. an increased autophagic flux in DMSC.

To confirm the increase in the autophagic flux of DMSC, we analyzed other indicators of autophagy in 72 h MPA-treated samples (Fig. 5c). Untreated and MPA-treated lysates were immunoblotted for SQSTM1/p62 which, acting as a link between LC3 and ubiquitinated substrates, is degraded by autophagy so that the p62 amount is considered to inversely correlate with autophagic activity. Figure 5c shows that the MPA treatment decreased p62 in DMSC as a consequence of faster autophagic flux. Likewise, the mammalian target of rapamycin (mTOR) and its downstream target, the ribosomal subunit p70S6 kinase 1 (S6K), were analyzed by *western blot*. These proteins are key players in nutrient sensing, promoting anabolic processes that lead to cellular growth. Under diverse stressful conditions, mTOR is inhibited, leading to the induction of autophagy. As shown in Fig. 5c, the active phosphorylated forms of these proteins were downregulated by MPA treatment.

### MPA induces premature senescence in DMSC cultures

**Fig. 6:**
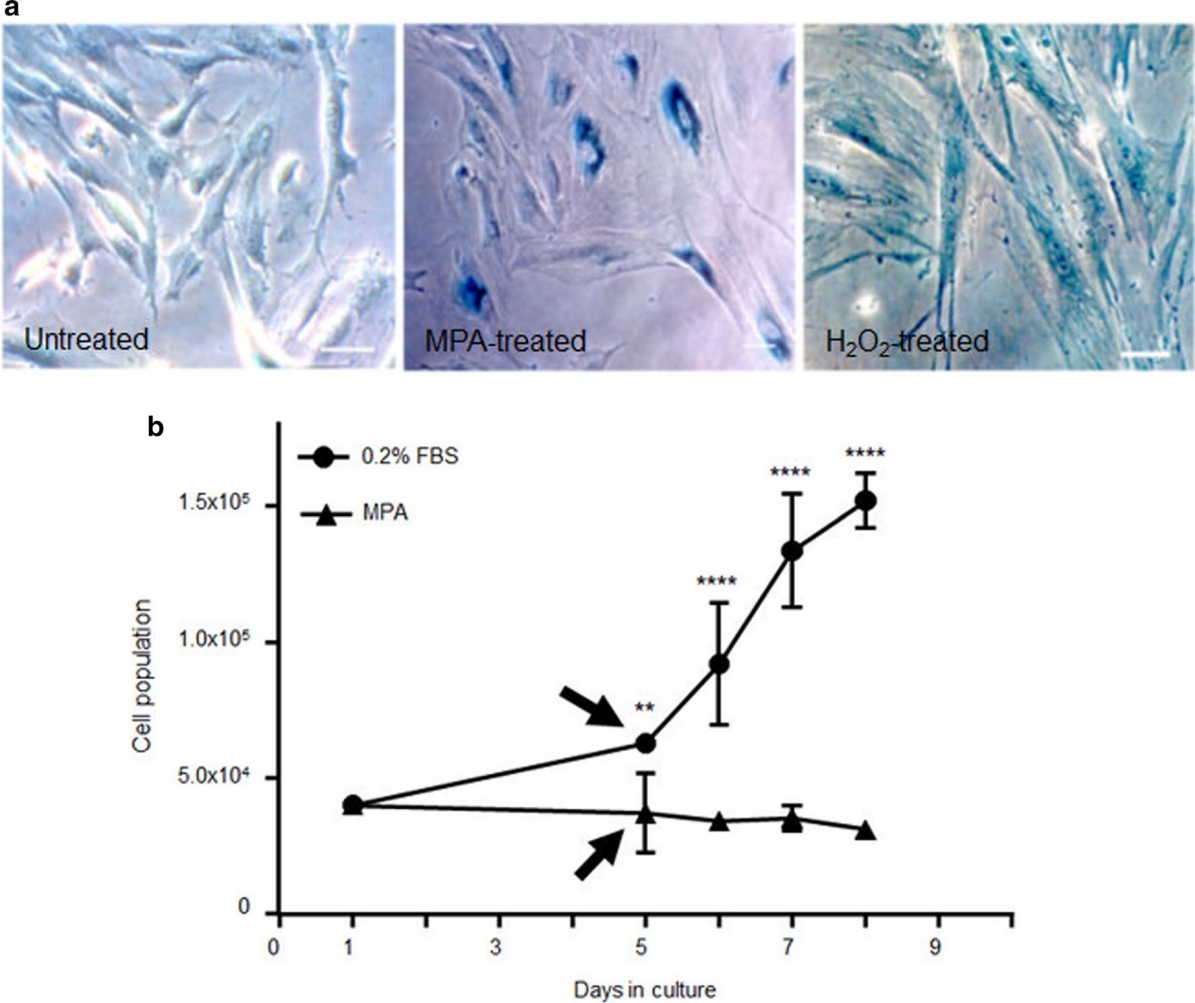
Senescence features and irreversible growth detention during culture of DMSC in the presence of MPA. **a** Photomicrograph of blue-colored staining for SA-β-gal activity in low confluency DMSC cultured with MPA for 72 h. Cells exposed to sublethal dose of H_2_O_2_ were used as positive control. H_2_O_2_ at a concentration of 250 µM was added to the culture for 4 h, then washed and cells incubated in fresh medium for additional 72 h. Untreated DMSC display a fibroblast-like morphology. MPA treated cells became larger in size and more flattened. **b** Growth kinetics of cultured DMSC. At day 1, cells were plated in 24-well plates in complete medium (10% fetal bovine serum) and 6 h later cells were arrested either by replacing the medium by 0.2% serum or by adding MPA. After 4 days, cells were washed, and then cultured in 10% serum-containing medium (arrow). Daily, cells were harvested and counted and the accumulated population was calculated (**P ≥ 0.01****P ≥ 0.0001)

Since MPA treatment does not induce apoptosis in DMSC (Fig. 1), we used SA-β-gal staining to assess whether the growth arrest caused by MPA on DMSC (Fig. 2) could result in premature senescence of the cell culture. DMSC treated with sublethal doses of H_2_O_2_ were used as a positive control of senescence. The photomicrographs of Fig. 6a show SA-β-gal positive cells in MPA- and H_2_O_2_-treated cultures. Morphological changes compatible with senescence were evident in both treatments when compared with untreated cells. Flattened and enlarged cells, as well as, the presence of actin stress fibers were visible, in both, MPA- and H_2_O_2_-treated DMSC.

Furthermore, to confirm the occurrence of a senescence process, we wanted to know whether MPA-induced growth arrest was an irreversible process (senescence) or, conversely, could be reversed by withdrawal of MPA (quiescence). As Fig. 6b shows, cells arrested by serum starvation and then stimulated with serum were able to resume proliferation, whereas MPA-arrested cells were unable to proliferate after MPA was withdrawn (Fig. 6b). These results suggest that MPA induces senescence in DMSC, i.e. an irreversible growth arrest that is different from quiescence, a reversible growth arrest induced by serum deprivation.

### Blockage of autophagy induces apoptosis to MPA-treated cells

**Fig. 7:**
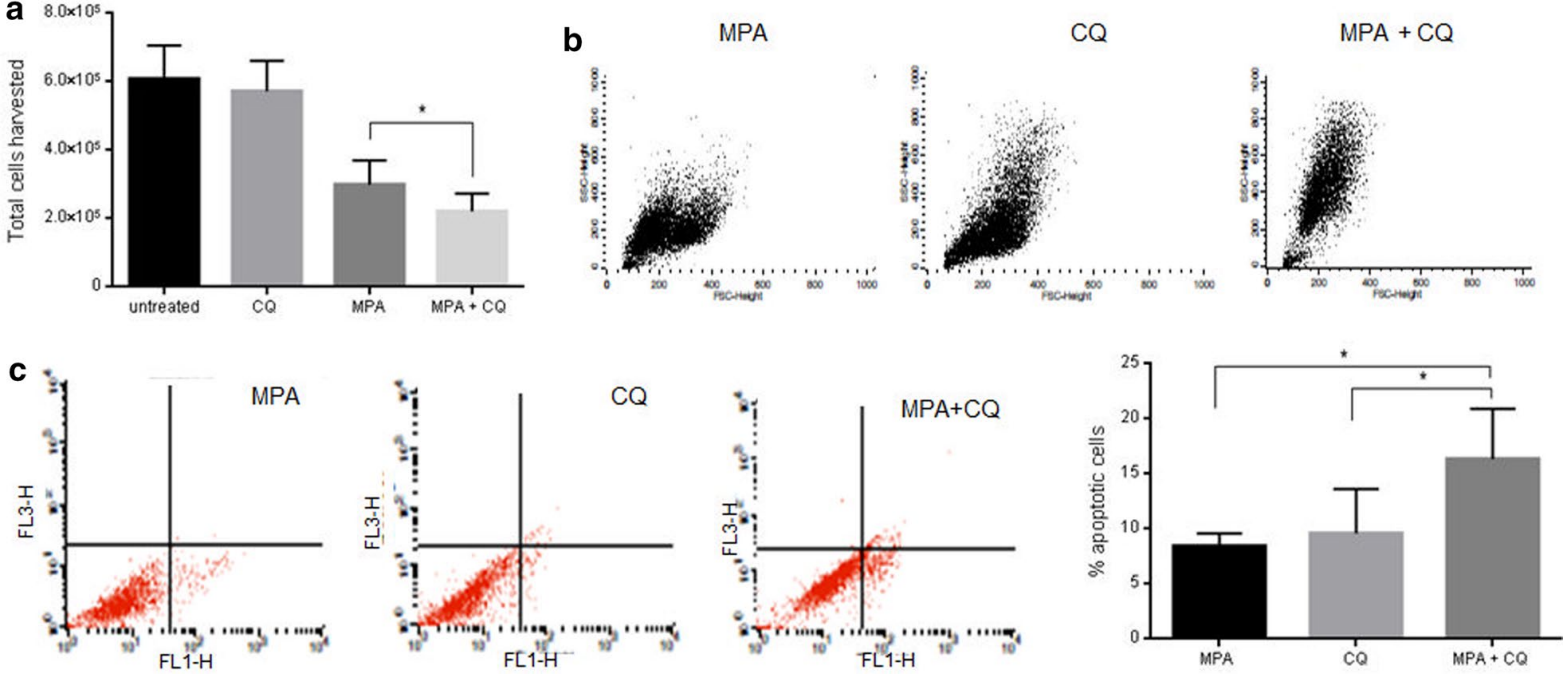
Consequences of autophagy blockage on MPA-treated DMSC. Cells plated the day before were exposed to 60 µM chloroquine (CQ) for 3 h. After CQ withdrawn, cells were treated with MPA for 72 h, and then the cells were harvested, counted and analyzed by flow cytometry. **a** MPA treatment after pretreatment with CQ significantly reduced the amount of harvested cells (*P ≥ 0.05). **b** Pretreatment with CQ induces morphological changes in MPA-treated DMSC. A smaller cell size (low FSC) with enlarged intracellular granularity (high SSC) is suggestive of early apoptotic events. **c** Annexin V staining reveals a significantly increase of the number of early apoptotic cells in MPA-treated cells when autophagy is impaired by CQ pretreatment (*P ≥ 0.05). These experiments were done in triplicate

To determine the role of autophagy in the MPA-mediated fate of decidual cells, DMSC were pretreated for 3 h with the autophagy inhibitor CQ before the MPA treatment. After 72 h, the cells were harvested and counted. As Fig. 7a shows, wells which have been treated with MPA after pretreatment with CQ showed a significant reduction in the number of cells respect to those treated with MPA alone. CQ alone did not affect the number of cells collected.

To assess whether the blockage of autophagy by CQ prior to MPA treatment could induce some form of cell death, DMSC were stained with annexin V and analyzed by flow cytometry. First, it was noted that DMSC exposure to MPA after CQ pretreatment changed their morphology shifting to a lower FSC (cell size) and a higher SSC (cell granularity), compared to MPA or CQ treatments alone (Fig. 7b), which could be compatible with the appearance of early apoptotic events. Indeed, the number of apoptotic DMSC after MPA treatment was significantly higher when autophagy was previously blocked by CQ (Fig. 7c).

## Discussion

The use of MPA during pregnancy to prevent allogenic organ rejection, or to treat diverse autoimmune diseases, has been associated with an increased risk of miscarriage during the first trimester and with a specific and consistent pattern of malformations in the newborn [31]. The mechanisms responsible of the increased risks of miscarriage by the use of MPA have never been addressed. Damage to placental components could be involved in this effect, and to the best of our knowledge there is not study addressing this issue. During pregnancy, the integrity of the feto-maternal interface is critical for the survival of the conceptus and for the maintenance of pregnancy. The decidua is the maternal component of the maternofetal interface. A crosstalk between the embryo and the decidua is established immediately post-conception contributing to either the development or the active rejection of the conceptus, and this mechanism of control is probably continued throughout the first trimester of pregnancy [32]. We hypothesized here that damage to the placental structures such as the decidua, might be involved in the increased risk of miscarriage induced by MPA.

This work analyzes the effects of MPA treatment on the biology of decidua mesenchymal stromal cells (DMSC) in order to identify potential mechanisms underlying miscarriage and fetal developmental failures. Treatment with a clinically relevant dose of MPA leads to a significant reduction in the amount of viable DMSC which cannot be attributed to an induction of cell death but the result of an anti-proliferative effect. The guanine nucleotides are building blocks of DNA and RNA, in addition to being involved in other relevant cellular processes. The availability of intracellular guanine nucleotides is regulated by the IMPDH-dependent guanine biosynthesis pathway. IMPDH exists in two isoforms, type I isoform that is constitutively expressed and is the predominant isoform in normal cells, and type II isoform that is highly expressed in proliferating cells, including lymphocytes and tumor cells [33]. IMPDH type II is the form mainly expressed in placental tissue [34] and is most potently inhibited by MPA [35].

In addition to the anti-proliferative effect, depletion of guanine nucleotides is also predicted to cause DNA damage. Lack of any type of nucleotide may increase the risk of misincorporation of deoxynucleotides into DNA during the S phase of the cell cycle as described elsewhere in the pyrimidine deficit [36]. DNA damage in normal cells triggers cell cycle arrest or causes cell death, preventing the duplication of the cells. As reported here, no apoptotic cells are found in MPA-treated DMSC cultures, although changes in the progression of DMSC through the cell cycle are detected. MPA treated cells tend to accumulate in late S and G2 phases without progression into the M phase, whereas a reduction of G0/G1 is detected. This pattern of cellular retention in G2 before entry into mitosis is suggestive of the onset of minor DNA damages during the replication phase [36]. The activation of the G2 checkpoint provides an opportunity for DNA repair. MPA treatment causes cell cycle arrest in G0/G1 in lymphocytes and vascular precursor cells [37]. The arrest of the cell cycle after DNA damage can also be induced at S or G2 phases, depending on the cell type, its active checkpoint mechanism, as well as its growth conditions [38]. We suppose that MPA induces a minor DNA damage in DMSC which cells try to repair before entering in apoptosis.

The nucleolus is the ribosome factory of cells and recently has also become the main cell stress sensor, with numerous evidence that connect the nucleolus with the cell cycle [39], even relating nucleolus stress to G2 arrest [40]. Because the size of the nucleolus correlates with the rate of ribosomal RNA (rRNA) synthesis, actively dividing cells often possess large nucleoli that ensure an optimum ribosome biogenesis and protein synthesis [27], whereas cell cycle arrest supposes a reduction in nucleolar size [41, 42]. In this work, actinomycin D, a selective inhibitor of mammalian rRNA synthesis, was used as a control of nucleolar disassembly, and the nucleolar protein nucleophosmin/B23 was used to detect the nucleoli. Our results showed that B23 was undetectable in actinomycin D treated cells while in MPA treated DMSC nucleoli appeared evidently disorganized and smaller than in untreated cells. The decrease in B23 fluorescence in nucleoli could be suggestive of a shift of B23 protein from the nucleolus to the nucleoplasm as described in cells exposed to several cytotoxic agents [43]. In addition, MPA treatment of DMSC strongly inhibited the synthesis of the 45S rRNA which serves as the precursor for 28S, 18S and 5.8S rRNAs. Given the very high G + C content (~ 70%) in human rDNA genes, inhibition of their expression is expected in guanine depletion caused by MPA. Lymphocytes and leukemic cell lines have an early and near-complete reduction of the 45S precursor rRNA, and translocation of nucleolar proteins from the nucleolus to the nucleoplasm after MPA exposure [44].

The nucleolus translates cellular stress signals into a cellular response by the stabilization of the tumor suppressor p53 [45]. Under normal cellular growth conditions, p53 levels are kept low by the action of the Hdm2 protein which is a homolog to Mdm2 (mouse double minute 2), a nucleolar protein involved in the nuclear export and proteasomal degradation of p53 [46]. Nucleolar impairment determines that p53 can no longer be degraded as the p53–Mdm2 interaction is disrupted. As reported here, treatment of DMSC with MPA induced nucleolar disruption and an increase in p53 protein levels as compared to untreated cells. p53 orchestrates different cellular responses, such as apoptosis, DNA repair, cell cycle arrest, senescence, metabolic adaptation, or autophagy, depending of the cell type and the cellular context [47]. Cell cycle arrest is achieved by diverse downstream effectors of p53 such as the protein p21 which acts as a potent cyclin-dependent kinase inhibitor (CDKI) regulating cell cycle progression at both G1, and G2 phases [48]. Our results show that MPA induced a robust increase in the p21 protein levels in DMSC. Increased p21 protein by MPA treatment resulting in cell cycle arrest has been reported in insulin-secreting cells [49] and in vascular precursor cells [37]. According to these reports, it is feasible to suggest that the tandem p53/p21 is the MPA mediator of cell cycle arrest in DMSC.

Autophagy is an additional mechanism induced in response to stressful conditions to preserve cellular viability. p21 and other CDKIs have been reported to induce autophagy [50], and reciprocally, the products of autophagy-related genes have been shown to trans-activate p21 [51]. We have shown in this report that MPA treatment of DMSC induced an autophagic degradative response. Increased LC3-II protein levels were detected in DMSC after 12 or 24 h of MPA-treatment indicating a greater amount of autophagosomes in the cells. A subsequent apparent decrease in LC3-II levels was suggestive of either a depletion of autophagic mechanisms, or a faster autophagic flux with LC3-II consumed at a very high rate. When analyzed over time LC3 levels in the presence of the autophagy inhibitor chloroquine, the number of autophagosomes was steadily increasing in MPA treated DMSC suggesting an increased autophagic flux. This conclusion is supported by the lower levels of SQSTM1/p62, phosphorylated mTOR and p70-S6K in MPA-treated cells. The role of autophagy as a homeostatic mechanism probably acquires great relevance in pregnancy. Autophagy is needed for the formation and maintenance of the placenta, and an inhibition of autophagy results in abnormal decidualization [52]. It seems well founded that any form of deregulation of the autophagic mechanism can have deleterious consequences. Upregulated autophagy with an increased expression of LC3-II protein has been described in placentas of women with severe preeclampsia [53], and in placentas of fetuses with intrauterine growth restriction [54]. Dysregulation of placental autophagy could also cause deleterious effects on embryos. Thus, autophagy activation in choriocarcinoma cells BeWo, used as an in vitro model of the placental barrier, triggers DNA damage on co-cultured neural precursors [55].

MPA-treated DMSC display features of senescent cells appearing larger than untreated cells, with flattened morphology and marked actin stress fibers, and increased SA-ß-galactosidase activity. These treated cultures were unable to resume normal proliferation after MPA removal, by contrast to what happened in the reversible quiescent state induced by serum starvation. Senescence is the state of irreversible cell-cycle arrest, which is part of the normal cellular aging process and a response to a variety of stresses. MPA induce senescence in chronic myeloid leukemia cell lines [56], vascular precursor cells [37], and DMSC, as we reported here. The role of senescence as a general response to physiologic stress seems increasingly important. By exiting the cell cycle, senescence limits the replication of old or damaged cells. Many studies have revealed the role of the p53/p21 signaling pathway in senescence by promoting cell growth arrest [57]. Evidence of a physiological ageing of placenta throughout gestation was first described in animal models and later in human placentas [58]. Placenta ageing acts as a contributor to labor inducing signals, and term placentas display molecular markers of cellular senescence such as SA-β-gal, an increased expression of the CDK inhibitors p16 and p21, and the tumor suppressor p53 [59]. Senescence markers are also elevated in placentas from unexplained stillbirth, and early-onset preeclampsia, suggesting an accelerated aging process in placentas of pathological pregnancy conditions [60]. Concerning decidua, a recently published study has found a correlation between excessive decidual senescence and recurrent pregnancy loss [61]. Decidual stromal cells affect the pregnancy microenvironment because of their role on the recruitment, distribution, and function of immune cells, as well as on tissue remodeling [3]. Arrested decidual growth can severely affect the biological role of the decidua. In addition, senescent decidual cells could promote senescence in neighboring placental tissues resulting in tissue dysfunction. As shown here, decidual stromal cells seem to be irreversibly damaged by MPA owing to the permanent cell cycle arrest which leads to a premature senescence. The maintenance of the DMSC senescent state probably is supported by an increase of the autophagic activity. Indeed, the blockage of autophagy by CQ appears to change the fate of DMSC following MPA treatment, from senescence where cells are still viable, toward cell death by apoptotic mechanisms. These results suggest an association between the DMSC senescent state and the induction of autophagy. DMSC are able to activate the autophagy as a rescue mechanism in response to guanine depletion supporting a senescence state and avoiding apoptosis. Although autophagy initially functions as a cellular recovery mechanism, it is possible that once a certain threshold is exceeded, autophagy may lead to the death of DMSC [62]. The exhaustion of decidual tissue because of the MPA effects would appear to be incompatible with maintaining pregnancy and would likely act as a promoter of preterm labor. Future studies on decidual cells from animal models of miscarriage by MPA should be carried on to consolidate this hypothesis.

## Conclusion

In the present study, we found that depletion of guanine nucleotides by MPA treatment of decidua mesenchymal stromal cells resulted in a severe reduction in pre-rRNA synthesis and disruption of the nucleolus. Ribosomal stress producing p53 stabilization led to p53-dependent p21 –mediated cell cycle arrest in late S and in G2 phases without progress to mitosis, which resulted in loss of proliferation capacity and decrease in viability of DMSC cultures. In the absence of an apoptotic response, decidua stromal cells activated mechanisms of autophagy and senescence. Placental trophoblasts and decidual cells mediate the active production of enzymes and hormones necessary for the maintenance of pregnancy, fetal growth and development. Any alteration in these cells, as that induced by MPA treatment, could cause loss of function and, consequently, produce miscarriage or detrimental effects on the fetus.

## Data Availability

The data used and analyzed during the current study are available on reasonable request to the corresponding author.

## Abbreviations

MPA: Mycophenolic acid
IMPDH: Inosine-5′-monophosphate dehydrogenase
DMSC: Decidua mesenchymal stromal cells
PBS: Phosphate-buffered saline
BrdU: 5-Bromo-2-deoxyuridine
PI: Propidium iodide
DAPI: 4′,6′Diamidino-2-phenylindole
CTCF: Corrected Total Cell Fluorescence
RT-qPCR: Real-time quantitative PCR
AD: Actinomycin D
LC3: Microtubule-associated protein 1A/1B-light chain 3
mTOR: Mammalian target of rapamycin
S6K: Ribosomal subunit p70S6 kinase 1
rRNA: Ribosomal RNA
Mdm2: Mouse double minute 2
CDKI: Cyclin-dependent kinase inhibitor
CDKs: Cyclin dependent kinases
CQ: Chloroquine
